# Metacognitive beliefs in individuals at risk for psychosis: a systematic review and meta-analysis of sex differences

**DOI:** 10.1007/s40211-020-00348-8

**Published:** 2020-04-27

**Authors:** Josef Baumgartner, Zsuzsa Litvan, Marlene Koch, Barbara Hinterbuchinger, Fabian Friedrich, Lukas Baumann, Nilufar Mossaheb

**Affiliations:** 1 Department of Psychiatry and Psychotherapy, Clinical Division for Social Psychiatry, Medical University of Vienna, Waehringer Guertel 18–20, 1090 Vienna, Austria; 2 Center for Medical Statistics, Informatics and Intelligent Systems (CeMSIIS), Institute for Medical Statistics, Medical University of Vienna, Spitalgasse 23, 1090 Vienna, Austria

**Keywords:** Metacognition, Psychosis, Sex, Metacognitive beliefs, High-risk, Prodrome, Metakognition, Psychose, Geschlecht, Metakognitive Überzeugungen, Hochrisiko, Prodromi

## Abstract

**Background:**

Sex differences were found in several domains in individuals at ultra-high risk for psychosis, but no previous work has systematically reviewed and analysed possible sex differences in metacognition in this population. However, alterations in metacognitive beliefs have been shown in the at-risk mental state for psychosis population. Our aim was to qualitatively review and quantitatively analyse the existing literature for data on sex differences in metacognitive beliefs—mainly depicted by the Metacognitions Questionnaire (MCQ) and its short form (MCQ-30)—in individuals with at-risk mental states.

**Methods:**

We performed a systematic review of the literature on metacognition in help-seeking adolescents and young adults at ultra-high risk for psychosis. We included peer-reviewed articles that included a high-risk for psychosis group assessed with operationalised criteria and instruments. For the quantitative meta-analysis, only studies comparing MCQ data in high-risk individuals were included. A fixed-effect meta-model was used and forest plots drawn for each subscale and overall score. The studies were weighted according to the inverse variance method in order to calculate pooled confidence intervals and *p* values.

**Results:**

No article on metacognitive beliefs in individuals at increased risk for psychosis explicitly reported possible sex differences. Our meta-analysis of 234 (57% male) individuals’ scores in the MCQ yielded no significant sex difference.

**Conclusions:**

Currently, no sex differences in metacognition can be described in the at-risk population; however, data are insufficient and heterogeneous with regard to thoroughly answering the question whether sex differences in clinical high-risk populations are mirrored in the metacognitive domain.

## Abstract (de)

**Grundlagen:**

Geschlechtsunterschiede wurden für Psychose-Risiko-Individuen in verschiedenen Bereichen gefunden, jedoch wurde Metakognition in dieser Population bisher nicht systematisch auf Unterschiede untersucht. Sehr wohl wurden Auffälligkeiten in metakognitiven Überzeugungen in Kohorten mit ultra-high-risk für Psychosen beschrieben. Wir wollten die aktuell existierende Literatur für Befunde über Geschlechtsdifferenzen in metakognitiven Überzeugungen – hauptsächlich abgebildet durch das Metacognitions Questionnaire (MCQ) und seine Kurzform (MCQ-30) – in Psychose-Risiko-Individuen qualitativ beschreiben und im Sinne einer Metaanalyse quantitativ untersuchen.

**Methodik:**

Wir vollführten einen systematischen Literaturüberblick über Metakognition in hilfesuchenden Jugendlichen und jungen Erwachsenen mit Psychose-Risiko. Dabei berücksichtigten wir „peer-reviewed“ Artikel, in denen Kohorten mit high-risk für eine Psychose eingeschlossen und mit standardisierten Kriterien und Instrumenten beurteilt wurden. Für die quantitative Metaanalyse inkludierten wir lediglich Studien die den MCQ verwendeten. Verwendet wurde ein Metamodell mit fixen Effekten, für jede Subskala und den Gesamt-Score wurden Forest-Plots erstellt. Die Studien wurden nach der Inverse-Varianz-Methode gewichtet, um gepoolte Konfidenzintervalle und *p*-Werte zu berechnen.

**Ergebnisse:**

Kein Artikel über metakognitive Überzeugungen in Individuen mit Psychose-Risiko berichtete explizit über mögliche geschlechtsbezogene Unterschiede. Die Metaanalyse aus MCQ-Punktwerten von 234 Individuen (57 % männlich) ergab keine geschlechtsbezogenen Unterschiede.

**Schlussfolgerungen:**

Bis dato können keine geschlechtsspezifischen Unterschiede in Metakognition in der Psychose-Risiko-Population beschrieben werden. Doch die Datenlage ist zu unzureichend und zu heterogen, um ausreichend genau zu beantworten, ob geschlechtsbezogene Unterschiede in Psychose-Risiko-Populationen vielleicht ihren Ursprung im metakognitiven Bereich haben.

## Introduction

Metacognition is generally described as “thinking about one’s own thinking” [1]. Metacognitive beliefs and dysfunctions have gained research focus in the past two decades. Their potential relevance with respect to the maintenance and even induction of symptoms in different psychiatric diseases such as psychotic disorders, anxiety disorders and depression [2, 3] is being increasingly discussed. It is speculated that the appraisal of anomalous experiences could play a critical role in the development of psychosis [4, 5]. As a matter of fact, metacognitive beliefs have been shown to be significantly altered in patients with psychotic disorders compared to healthy controls [6]. Furthermore, a recent meta-analysis has provided evidence for significant differences in metacognitive beliefs in individuals in an at-risk mental state for psychosis (ARMS) [7]. The ARMS concept identifies young people at markedly increased risk for psychosis [8]. It is defined as experiencing at least one of the three ultra-high risk (UHR) criteria: (i) genetic risk and deterioration syndrome, i.e. genetic risk for psychosis in a first-degree relative or schizotypal disorder in the individual and relevant drop in functioning; (ii) attenuated psychotic syndrome, and (iii) brief limited intermittent psychotic symptoms.

Sex aspects play an important role in individuals in an ARMS. Similarly to findings in patients with established diagnosis of schizophrenia [9], sex differences in UHR individuals with respect to poorer mentalizing abilities, more serious negative symptoms, higher substance abuse comorbidity and poorer social functioning have been described, with men showing more pronounced alterations compared to women [10]. However, sex differences in metacognitive beliefs have not been previously described in the ARMS population.

Most studies on metacognition examined metacognitive beliefs, a specific aspect of metacognition, designating beliefs about the perceived importance of directing or controlling one’s own cognitive processes [7]. Metacognitive beliefs have been shown to lead to attention and cognitive processing biases [11]. The most commonly used instrument for the assessment of metacognitive beliefs to date is the Metacognitions Questionnaire (MCQ), which was developed on the basis of the Self-Regulatory Executive Function model to measure dysfunctional metacognitive beliefs [12]. The original version of the MCQ consists of 65 items; however a shorter version (MCQ-30) with 30 items is also available [13]. Both versions have 5 subscales, each assessing one dimension of dysfunctional metacognitive beliefs: (1) positive beliefs about worry (e.g. ‘Worrying helps me to get things sorted out in my mind’), (2) negative beliefs about uncontrollability of thoughts and corresponding danger (e.g. ‘Worrying is dangerous for me’ and ‘I cannot ignore my worrying thoughts’), (3) cognitive confidence (e.g. ‘I have a poor memory’), (4) negative beliefs about thoughts in general, including themes of responsibility, punishment and superstition (e.g. ‘If I did not control a worrying thought, and then it happened, it would be my fault’) and (5) cognitive self-consciousness (e.g. ‘I pay close attention to the way my mind works’).

The original MCQ was developed in 1997 and its validation study showed significantly lower scores for healthy women in the subscales ‘negative beliefs about thoughts in general’ and ‘cognitive self-consciousness’ compared to healthy men [12]. The primary validation study for MCQ-30 revealed non-significantly higher scores for healthy males in subscales ‘cognitive confidence’ and ‘cognitive self-consciousness’ and in overall scores compared to healthy females [13]. The validation study of the Greek translation of the MCQ-30 found that women scored significantly higher than men in the overall MCQ-30 and the ‘negative beliefs about uncontrollability of thoughts and corresponding danger’ (2) and ‘negative beliefs about thoughts in general’ (4) subscales [14]. In the validation study of the Spanish version of the MCQ-30 on the other hand men had significantly higher results in the subsections ‘positive beliefs about worry’ (1) and ‘negative beliefs about thoughts in general’ (4) [15].

Metacognitive beliefs are, however, not the only aspect of metacognitive functioning investigated in the ultrahigh risk for psychosis population. As a matter of fact, global metalevel of performance monitoring the correctness of cognitive tasks has been used as a proxy for metacognitive functioning [16]. Furthermore, Buchy et al. [17] depict four components of metacognition (self-reflectivity, understanding others’ minds, decentration and mastery) using the Metacognition Assessment Scale (MAS-A) [18]. Eisenacher and her colleagues [19] examined metamemory functioning by investigating performance in memory monitoring in individuals with ARMS.

No previous work has systematically reviewed and analysed whether alterations in metacognitive beliefs differ between male and female UHR individuals.

## Aim

The goal of our work was to qualitatively review and quantitatively analyse the existing literature for data on sex differences in metacognitive beliefs—mainly depicted by the MCQ/MCQ-30—in UHR individuals.

## Methods

Two researchers independently performed a systematic literature search following the PRISMA guidelines [20]. The search was conducted on July 28, 2018, in Ovid MEDLINE, PsycINFO, EBM Reviews, PSYNDEX, Scopus and CINAHL databases applying the search term “metacogn*” in combination with “at-risk mental state” or “ARMS” or “ultra-high risk” or “UHR” or “clinical high risk” or “CHR” or “prodrom*” or “psychosis” or “psychotic” or “schizophren*”. Moreover, studies’ reference lists were manually searched for further relevant studies. In order to be included in the qualitative analysis studies had to be published in a peer-reviewed journal, and examine a UHR for psychosis group assessed with operationalised criteria and instruments. For the quantitative meta-analysis of potential sex differences, only studies comparing MCQ/MCQ-30 data in UHR individuals were included. Authors of the eligible studies were contacted for further data. The two researchers screened the studies independently for eligibility; thereafter unclear cases were discussed. Information and data of each eligible study were gathered with the help of a predetermined data extraction sheet.

### Statistical analysis

Due to the limited number of studies, a fixed-effect meta model was used instead of a random effect model, as no conclusive value could be expected from computing a heterogeneity coefficient [21]. Forest plots were drawn for each subscale and for overall scores. The studies were weighted according to the inverse variance method in order to calculate pooled confidence intervals and *p* values.

## Results

### Literature search

**Fig. 1:**
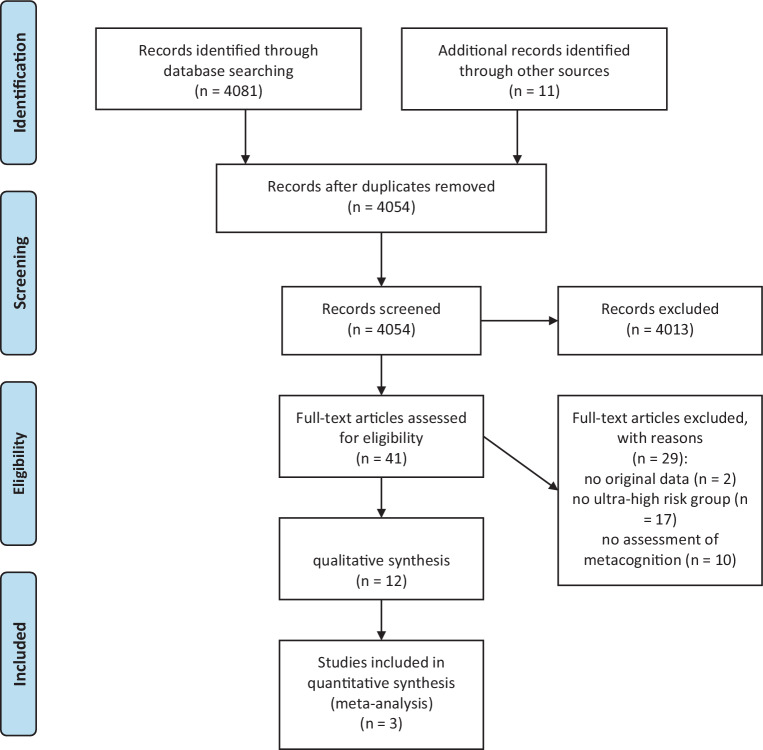
PRISMA 2009 flow diagram

Figure 1 depicts the PRISMA flow diagram highlighting the assessment of the literature for eligibility for qualitative and quantitative analysis respectively.

### List of 12 studies eligible for qualitative analysis (Table 1)

**Table 1:** Studies in qualitative analysis

Author, year	*n* ARMS	ARMS screening instrument	Control group(s) (CG) (type)	*n* control group	Metacognition instrument
Morrison et al., 2002 [22]	31	PANSS	Healthy CG	50	MCQ
Morrison et al., 2006 [23]	58	PANSS	Healthy CG (high caseness)	56	MCQ
Morrison et al., 2007 [24]	43	PANSS	Healthy/Psychosis CGs	188/73	MCQ
Brett et al., 2009 [27]	32	CAARMS	Healthy/Psychotic-like experiences/Psychosis CGs	32/24/27	MCQ
Barkus et al., 2010 [25]	45	PANSS	Healthy/High schizotypy/Trait and state CGs	80/23/18	MCQ
Palmier-Claus et al., 2013 [36]	27	CAARMS	No CG	–	MCQ-30
Barbato et al., 2014 [28]	153	SIPS	Help-seeking CG	68	MCQ
Welsh et al., 2014 [29]	31	CAARMS	Healthy CG	76	MCQ-30
Scheyer et al., 2014 [16]	19	SIPS	Help-seeking CG	39	Novel metacognitive approach
Morrison et al., 2015 [26]	117	CAARMS	Help-seeking CG	318	MCQ-30 (only 3 subscales)
Buchy et al., 2015 [17]	29	SIPS	No CG	–	Meta-cognitive Assessment Scale (MAS)
Eisenacher et al., 2015 [19]	34	Early Recognition Inventory	Healthy/FEP CGs	38	Metamemory with DRM paradigm

As described by Cotter et al. [7], several of the above-mentioned studies used overlapping ARMS cohorts. Specifically, cohorts in the three papers by Morrison et al. [22–24] as part of the EDIE‑I trial and cohorts in the papers by Barkus et al. [25] and Morrison et al. [26] as part of the EDIE-II trial had overlapping participants.

### Qualitative analysis

None of the 12 studies reported sex differences in their original publications. That is why we reached out to the authors of the studies used for the quantitative analysis (9 of the original 12 publications used MCQ as an assessment instrument and were thus found to be eligible for quantitative comparison) to gather additional, unpublished data regarding sex distribution. These 9 publications report data of 6 study cohorts. We received the requested additional data from 3 of these 6 study cohorts, and we were able to collect overall and subscores data of the MCQ/MCQ-30 from 234 (134 male and 100 female) UHR individuals (Table 2; [27–29]). All studies matched ARMS groups to control groups regarding age and sex. To our knowledge, to date it has not been tested whether sex influences metacognitive beliefs measured with the MCQ/MCQ-30 in ARMS populations.

**Table 2:** Demographic data of included studies

	Brett et al., 2009 [27]	Barbato et al., 2014 [28]	Welsh et al., 2014 [29]
*n* (ARMS group)	32	171	31
Mean age (SD)	24.3 (3.6)	19.7 (4.2)^a^	15.3 (1.4)
Age range, years	20–33	Not available	12–17
Male sex	21 (66%)	98 (57%)	15 (48%)

^a^mean age and standard deviation (SD) for original study cohort of 171 participants

*ARMS* individuals in an at-risk mental state for psychosis

### List of 5 studies eligible for quantitative analysis (Table 3)

**Table 3:** Studies in quantitative analysis

Author + year	*n* ARMS	ARMS screening instrument	Control group(s) (type)	*n* control group	Metacognition instrument
Brett et al., 2009 [27]	32	CAARMS	Healthy/Psychotic-like experiences/Psychosis CGs	32/24/27	MCQ
Barbato et al., 2014 [28]	153 (*n* = 171 upon written request)	SIPS	Help-seeking CG	68	MCQ
Welsh et al., 2014 [29]	31	CAARMS	Healthy CG	76	MCQ-30
Morrison et al., 2006^a^ [23]	58	PANSS	Healthy CG (high caseness)	56	MCQ
Morrison et al., 2015^a^ [26]	117	CAARMS	Help-seeking CG	318	MCQ-30 (only 3 subscales)

^**a**^data not available

### Quantitative analysis

**Fig. 2:**
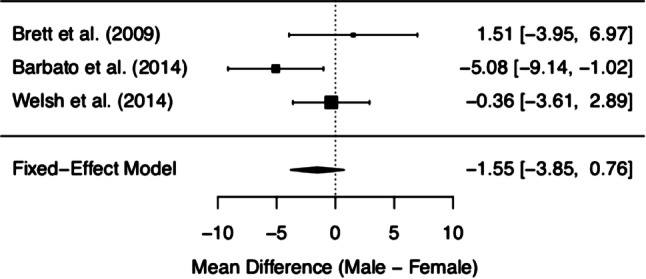
Positive beliefs about worry (Metacognitions Questionnaire subscale 1)

**Fig. 3:**
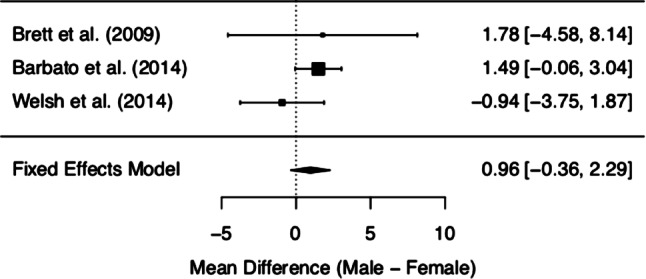
Negative beliefs about uncontrollability and danger (Metacognitions Questionnaire subscale 2)

**Fig. 4:**
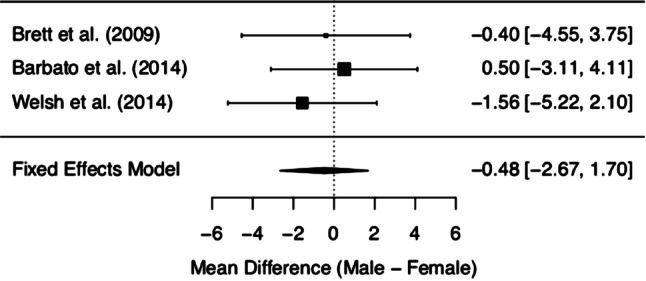
Cognitive confidence (Metacognitions Questionnaire subscale 3)

**Fig. 5:**
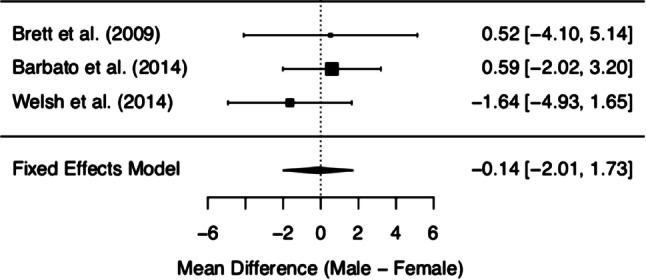
Negative beliefs about responsibility and superstition (Metacognitions Questionnaire subscale 4)

**Fig. 6:**
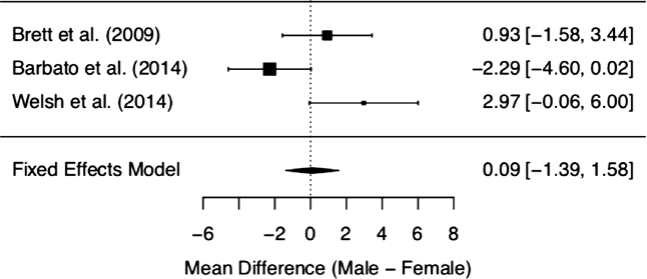
Cognitive self-consciousness (Metacognitions Questionnaire subscale 5)

**Fig. 7:**
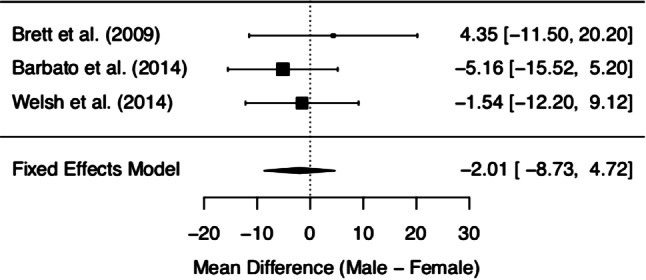
Overall Metacognitions Questionnaire score

MCQ/MCQ-30 subscores and overall score of the three included studies are depicted in Figs. 2, 3, 4, 5, 6 and 7. Fig. 2 shows “positive beliefs about worry” (MCQ subscale 1), Fig. 3 “negative beliefs about uncontrollability and danger” (MCQ subscale 2), Fig. 4 “cognitive confidence” (MCQ subscale 3), Fig. 5 “negative beliefs about responsibility and superstition” (MCQ subscale 4), Fig. 6 “cognitive self-consciousness” (MCQ subscale 5), and Fig. 7 overall scores.

## Discussion

Our meta-analysis of the available data on metacognitive beliefs in UHR individuals revealed no statistically significant differences in overall scores between males and females. To our knowledge, this is the first systematic review on sex differences in metacognition and metacognitive beliefs measured by different instruments.

Although the overall finding shows no differences between women and men at UHR for psychosis in metacognitive beliefs, a closer look at the individual data is worthy. The subsection on ‘positive beliefs about worry’ yielded significant results in one study [28], with higher scores in UHR women. However, upon addition of the remaining data, this difference diminished. The same effect was found in the subscale ‘cognitive self-consciousness’ with higher scores in females in the study by Barbato et al. [28], but lower scores in women in the study by Welsh et al. [29]. These effects might be due to differences in study samples and methodology of the included studies. As a matter of fact, the data made available derives from three heterogeneous studies with samples differing in several basic characteristics (see the *Quantitative results* section Figs. 2–Fig. 7 showing demographic data). Mean age was quite disparate, i.e. Brett et al. [27] included adults only, whereas Welsh et al. [29] assessed only youths between the age of 12 and 17. While these two latter studies had smaller sample sizes (around 30 participants each), three-quarters of the data we analysed was derived from Barbato et al. [28] with a sample size of 171 ARMS individuals. Accordingly, the latter will be expected to most significantly influence the result of our meta-analysis. Indeed, the participants in the study by Barbato et al. [28] were older than those in the study by Welsh et al. [29] (mean age of 19.7 years versus 15.3 years). With respect to metacognitive beliefs in younger age, a validation study for the children and adolescent version of the MCQ has shown the following: adolescents reported greater ‘cognitive self-consciousness’ (subscale 5) than children, and adolescent girls scored higher in overall MCQ than adolescent boys [30]. In the overall development in healthy individuals, metacognitive abilities increase over adolescence, peaking in adulthood, with healthy women showing better abilities than men [31]. Thus, the comparison of UHR populations at disparate ages might have biased our results. It is also noteworthy that recently the validity of the attenuated psychosis concept has been described as decreasing with younger age [32], possibly adding to the heterogeneity of the sample.

Though adequate efforts were made, we were not able to access all published MCQ data in UHR samples. Therefore, our analysis included MCQ data of around 60% of the over 400 ARMS individuals, whose data have been published.

With respect to operationalisation of ARMS status, the Barbato et al. study [28] is the only one that used the Structured Interview of Prodromal Symptoms (SIPS) as the standardized assessment, whereas the other studies used the Comprehensive Assessment of “At-Risk Mental State” (CAARMS); however we do not expect a major bias from this since definitions of UHR status are maintained in all studies.

While there has been great effort in the past 20 years to understand the metacognitive alterations in prodromal stages of psychosis [6, 7], only limited research emphasized sex-specific differences in metacognition. That is even in spite of the fact that validation studies in various languages recognized differences in metacognitive beliefs even in healthy participants [12–14]. In the last few years alterations in metacognition have not only been interpreted as characteristic markers for psychotic prodromes, but more generally as an important factor in inducing and aggravating psychiatric illness [33]. As previously discussed, there are increasing indications pointing towards the importance of metacognition in a broader aspect of psychologic functioning as well as psychiatric disease development and maintenance [33]. Alterations in metacognition are possibly decisive cognitive factors not only in psychotic episodes, but also in mood disorders and anxiety disorders [33]. On the other hand metacognitive biases could also be just a broad symptom of various psychiatric diseases and may also decline with remission of the respective episode.

Neurocognitive abilities have been shown to be a necessity for the functioning of social cognition and metacognition, but not solely [34]. The association between cognition and metacognitive performance in patients with schizophrenia spectrum disorder was found to grow weaker with increasing conceptual disorganization as measured in the Positive and Negative Syndrome Scale (PANSS); however this effect was not shown between neuro- and social cognition [35]. The studies included in this meta-analysis adjusted neither for neurocognitive nor for social cognition parameters, as these were not their primary markers.

## Conclusion

The potential differences between women and men at ultrahigh risk for psychosis with respect to metacognition have not yet been sufficiently examined. The heterogeneity of the sparse available data adds to the difficulties in interpreting the findings. Further research is warranted on this topic.
